# Real-Life Management of FLT3-Mutated AML: Single-Centre Experience over 24 Years

**DOI:** 10.3390/cancers16162864

**Published:** 2024-08-17

**Authors:** Saveria Capria, Silvia Maria Trisolini, Lorenzo Torrieri, Elena Amabile, Giovanni Marsili, Alfonso Piciocchi, Walter Barberi, Anna Paola Iori, Daniela Diverio, Daniela Carmini, Massimo Breccia, Maurizio Martelli, Clara Minotti

**Affiliations:** 1 Hematology AOU Policlinico Umberto I, 00161 Rome, Italy; trisolini@bce.uniroma1.it (S.M.T.); barberi@bce.uniroma1.it (W.B.); iori@bce.uniroma1.it (A.P.I.); diverio@bce.uniroma1.it (D.D.); d.carmini@policlinicoumberto1.it (D.C.); minotti@bce.uniroma1.it (C.M.); 2 Hematology, Department of Translational and Precision Medicine, “Sapienza” University, 00185 Rome, Italy; torrieri@bce.uniroma1.it (L.T.); amabile@bce.uniroma1.it (E.A.); breccia@bce.uniroma1.it (M.B.); martelli@bce.uniroma1.it (M.M.); 3 GIMEMA Foundation, 00182 Rome, Italy; g.marsili@gimema.it (G.M.); a.piciocchi@gimema.it (A.P.)

**Keywords:** acute myeloid leukemia (AML), FLT3 mutations, tyrosine kinase inhibitors (TKIs), midostaurin, complete remission (CR), hematopoietic stem cell transplantation (HSCT), minimal residual disease (MRD), relapse, chemotherapy, long-term outcomes

## Abstract

**Simple Summary:**

FLT3 mutations are common in acute myeloid leukemia (AML) and are often associated with poorer outcomes and higher relapse rates. This study examined the long-term outcomes of FLT3-mutated (FLT3m) AML patients who received intensive treatment at our institution over the past 24 years, comparing the results in patients who received standard chemotherapy alone with those who received a FLT3 inhibitor (FLT3i). Our single-center, real-life experience confirms the impact of FLT3i administration on survival, both in frontline and salvage settings. In addition, our results underline that the proper positioning of allogeneic stem cell transplantation in the therapeutic algorithm is still an unmet need. These findings suggest that incorporating FLT3-targeting agents into treatment plans may improve outcomes for AML patients while highlighting the need for the continued optimization of treatment strategies to enhance patient survival and quality of life.

**Abstract:**

We analyzed 140 patients with a median age of 51 years; 21% had WBC ≥ 100 × 10^9^/L, and 52% had an NPM1 co-mutation. Until 2018, 101 patients received chemotherapy; thereafter, 39 received 3+7+midostaurin. The overall CR rate was 64%, higher in NPM1 mutant patients (73%). Univariate analysis showed that NPM1 mutation (*p* = 0.032) and WBC < 100 × 10^9^/L (*p* = 0.013) positively influenced the response, with a trend for FLT3i administration (*p* = 0.052). Multivariate analysis confirmed WBC count as an independent prognostic factor (*p* = 0.017). In CR1, 41/90 patients underwent allogeneic and 18 autologous transplantation. The median EFS was 1.1 vs. 1.6 years in autografted and allografted patients, respectively (*p* = 0.9). The one-year non-relapse mortality was 0.00% for autologous and 28% for allogeneic transplants (*p* = 0.007); CIR at 1 and 3 years was higher in autologous transplants (39% vs. 15% and 57% vs. 21%, *p* = 0.004). The median survival was not reached in the FLT3i group. Overall, 69 patients received stem cell transplantation (18 autologous, 51 allogeneic). Post-transplant FLT3i was resumed in eight patients, all alive after a median of 65 months. Allogeneic transplantation is crucial in FLT3-mutated AML, but the next challenge will be to identify which patients can benefit from transplants in CR1 and in which to intensify post-transplant therapy.

## 1. Introduction

Approximately 30% of newly diagnosed acute myeloid leukemia (AML) patients exhibit activating mutations in FMS-related tyrosine kinase-3 (FLT3), with this incidence decreasing with age. FLT3 is a receptor tyrosine kinase expressed on hematopoietic stem cells and on myeloid and lymphoid progenitor cells. The mutations can be classified as either internal tandem duplications (ITD) at the juxta-membrane domain level or point mutations at the tyrosine kinase domain (TKD) level, the latter being less common. The FLT3 ligand is released by hematopoietic stem cells, lymphocytes, and cells that comprise the medullary stroma. Upon activation by the FLT3 ligand, the FLT3 receptor initiates signaling cascades through PI3K, STAT5, and RAS, thereby promoting cell growth and differentiation. The mutation of FLT3 results in the constitutive activation of the receptor and an increase in its responsiveness to the FLT3 ligand. Individuals with FLT3-ITD mutations typically present with a normal karyotype and a high disease burden at presentation [1].

FLT3 is a late event during leukemogenesis, and thus, FLT3-mutant (FLT3m) AML is a heterogeneous disease. It is often associated with nucleophosmin-1 co-mutation (NPM1m), which suggests that there is a molecular synergism in the leukemogenesis process. In comparison to wild-type FLT3 AML, FLT3m AML is associated with an increased risk of relapse and inferior survival (OS), particularly in patients without concomitant NPM1 mutation [2,3].

The introduction of FLT3 inhibitors (FLT3is) into clinical practice has represented a significant therapeutic breakthrough in the prognosis of FLT3m AML. This is evidenced by the improvement in OS demonstrated with intensive chemotherapy combined with midostaurin or quizartinib in newly diagnosed patients and with gilteritinib or quizartinib in relapsed/refractory (R/R) patients [3]. 

Midostaurin, a first-generation multi-targeted kinase inhibitor, is currently approved by the FDA and EMA for induction and consolidation therapy in patients with newly diagnosed FLT3m AML. A prospective, multinational, randomized trial (CALGB 10603-RATIFY) compared induction chemotherapy with the “3+7” regimen plus midostaurin versus placebo. The experimental arm demonstrated significantly longer overall survival (OS) and event-free survival (EFS) compared to the control arm. In comparison to first-generation inhibitors (midostaurin [4], sunitinib [5], lestaurtinib [6], sorafenib [7]), second-generation inhibitors (gilteritinib [8], crenolanib [9], quizartinib [10]) exhibit enhanced potency and selectivity for the FLT3 receptor while simultaneously exhibiting reduced toxicity.

Following the results of the phase III ADMIRAL trial, which randomized 371 patients with R/R FLT3m AML aged 18 years or older to reinduction therapy with oral gilteritinib versus standard chemotherapy, the FDA and EMA approved gilteritinib for this indication. The study demonstrated a significant survival benefit in the gilteritinib arm compared to chemotherapy (median OS 9.3 months vs. 5.6 months). Additionally, the tolerability of gilteritinib was found to be superior to that of chemotherapy [8].

Although initial remission rates are high with current therapies, relapse remains a significant limitation to long-term outcomes due to the emergence of resistance to FLT3i. The development of novel therapeutic strategies to be employed in conjunction with standard clinical practice in both frontline and R/R patients is of paramount importance in order to enhance outcomes.

The objective of this study was to, retrospectively, analyze the long-term outcomes of FLT3m AML patients who were eligible for intensive treatment and were referred to our institution since 1999. This study focused on changes in management over time and on the impact of tyrosine kinase inhibitor (TKI) treatment on long-term outcomes. 

## 2. Materials and Methods

### 2.1. Data Sources and Treatment Sequence

A comprehensive review of medical records and databases was conducted for all patients with FLT3m AML who were eligible for intensive chemotherapy and consecutively treated at the Hematology Department of our institution from October 1999 to June 2023. This observational study was conducted in accordance with the tenets of the Declaration of Helsinki and the privacy policy of the Policlinico Umberto I, Rome.

The fitness of patients for intensive induction therapy was evaluated using the Eastern Cooperative Oncology Group (ECOG) performance status at diagnosis and the SIE/SIES/GITMO fitness criteria [11]. Two distinct intensive regimens were employed: the DCE/DIA schedule for induction and consolidation [12] was employed until 2017; thereafter, all patients received 3+7/HD Ara-C in combination with midostaurin for both induction and consolidation, in accordance with the product label indications. For transplant-eligible patients, our institution’s historical approach is to proceed with allogeneic hematopoietic stem cell transplantation (allo-HSCT) in the first complete remission (CR1) after one or at least two consolidation cycles, contingent on donor availability.

Patients lacking a matched related donor and lacking FLT3 status information at the time were treated with autologous peripheral blood stem cell transplantation (ASCT). In more recent times, the indication for ASCT is restricted to a selected group of patients with NPM1m/FLT3 TKD at diagnosis and in molecular remission following consolidation. 

The conditioning regimen used for allo-HSCT was in most cases the TBF regimen [13]. MUD transplants received ATG, while haploidentical transplants were treated with post-transplant cyclophosphamide as a prophylactic measure against graft-versus-host disease (GVHD) [14]. For ASCT, the Bu-CY2 regimen was employed [15]. 

Patients who did not achieve CR after first-line treatment received a salvage chemotherapy regimen, in some cases combined with the TKI inhibitor sorafenib, while gilteritinib was used as a single agent as a bridge to transplantation since it became commercially available.

Patients with a persistent FLT3 mutation in complete hematological remission following allo-HSCT received preemptive TKI treatment for at least two years or until hematological relapse.

### 2.2. Diagnosis of FLT3/NPM1 Mutations

All patients underwent a determination of the their FLT3 ITD/TKD mutation status in bone marrow or peripheral blood leukemic cells collected at the time of initial presentation. Total RNA was extracted from Ficoll-Hypaque isolated mononuclear cells using the Maxwell^®^ RSC semi-automated instrument and the Maxwell^®^ RSC simplyRNA Blood Kit (Promega) in accordance with the manufacturer’s instructions. One microgram of total RNA was reverse-transcribed using Superscript IV VILO MasterMix (Life Technologies) in accordance with the manufacturer’s instructions. The amplification of the FLT3-ITD and NPM genes was conducted in accordance with the methodology proposed by Noguera et al. [16], and the resulting PCR product was sequenced using the standard Sanger method [17]. Fragment analysis was conducted via capillary electrophoresis on an ABI 3130 or ABI 3500 instrument, utilizing 1 μL and/or 1 μL of a 1:10 diluted PCR product, which was mixed in a solution of deionized formamide containing GeneScan 500 ROX dye size standard. The presence of the D835 mutation in the TKD domain was tested in accordance with the procedures outlined by Murphy et al. [18].

Patients with NPM1 mutations of types A, B, and D were monitored for MRD according to the procedures outlined by Gorello et al. [19]. In patients with wild-type NPM1, MRD was detected, whenever possible, by multicolor flow cytometry on whole bone marrow samples with a sensitivity of 10^−4^–10^−5^ [20] and a cut-off for positivity of 0.03%.

### 2.3. Statistical Analysis 

The characteristics of the patients were summarized using frequencies and percentages for categorical variables, whereas continuous variables were described using medians and their relative ranges. The associations between categorical variables were assessed using the chi-squared test and Fisher’s exact test. A two-tailed *p*-value of <5% was considered statistically significant. For multivariate analysis, the logistic regression model was used. OS and EFS were estimated using the Kaplan–Meier (KM) product limit method. Univariate comparisons were performed using the log-rank test. The cumulative incidence of relapse (CIR) and non-relapse mortality (NRM) were analyzed together using a competing risks model. Univariate comparisons were performed using the Gray test. Statistical analysis was performed with R version 4.2.2. 

## 3. Results

The study population consisted of a total of 140 patients (68 males and 72 females) who were consecutively admitted to our institution from October 1999 to June 2023. Patients’ demographics are presented in Table 1.

The median age at diagnosis was 52 years (range: 14–73), with 38 patients (27%) being ≥60 years of age. The median white blood cell (WBC) count was 50 × 10^9^/L (0.22–349). In 28 patients (21%), the WBC count exceeded 100 × 10^9^/L. CNS involvement was documented at diagnosis in 4 of the 82 evaluable patients.

The ITD/TKD ratio was 120/20, and the concurrent NPM1 mutation was observed in 52% of patients (73 individuals). The majority of patients (90%) exhibited intermediate-risk cytogenetics.

The induction treatment regimen consisted of standard chemotherapy in 101 patients (72%) (no-TKI cohort), while 39 patients received chemotherapy combined with midostaurin (TKI cohort). Seven patients died during the induction phase, all of whom were in the no-TKI cohort.

The overall CR rate was 64% (90/140), in particular 59.4% in the no-TKI group and 77% in the TKI group. In NPM1 mutant patients, the CR rate was 73% (53/73) irrespective of tyrosine kinase inhibitor (TKI) treatment during induction.

The CR rate was comparable between younger and older patients (67.6% vs. 55.2%—*p* = 0.4) and was positively correlated with the presence of a co-existing NPM1 mutation (*p* = 0.032) and a WBC count of less than 100 × 10^9^/L (*p* = 0.013). The administration of TKIs during the induction phase demonstrated a trend towards a significant effect on the achievement of complete remission (*p* = 0.052).

In a multivariate logistic regression model adjusting for sex and age, only WBC count was confirmed to have an independent prognostic impact on response (OR 3.04, CI: 1.23–7.71, *p* = 0.017) (Table 2).

**Figure 1:**
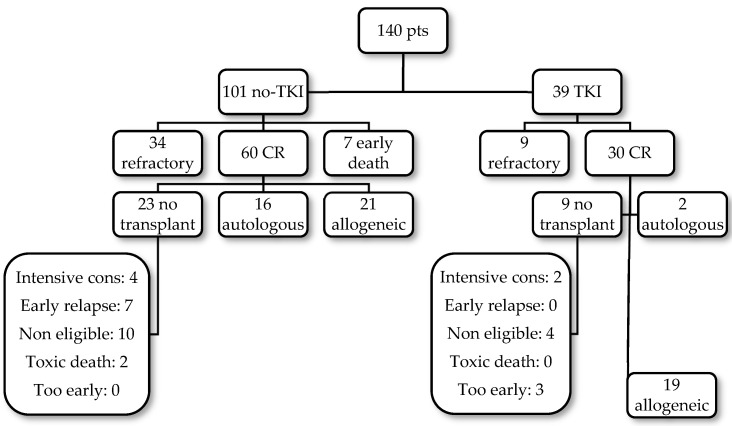
Patients’ disposition.

The disposition of patients after consolidation in both patient populations is shown in Figure 1. 

Allogeneic stem cell transplantation was performed in 40/90 patients who achieved CR1 (21/60 no-TKI and 19/30 TKI). Autologous transplantation was performed in 18 patients, 16 without TKIs and 2 with TKIs. The median interval from diagnosis to transplantation was 5 months (range 4–7). In 32 patients, no transplantation was performed. The transplantation rate between no-TKI and TKI patients is not statistically significant (*p* = 0.4). The reasons for not transplanting are shown in Table 3.

**Figure 2:**
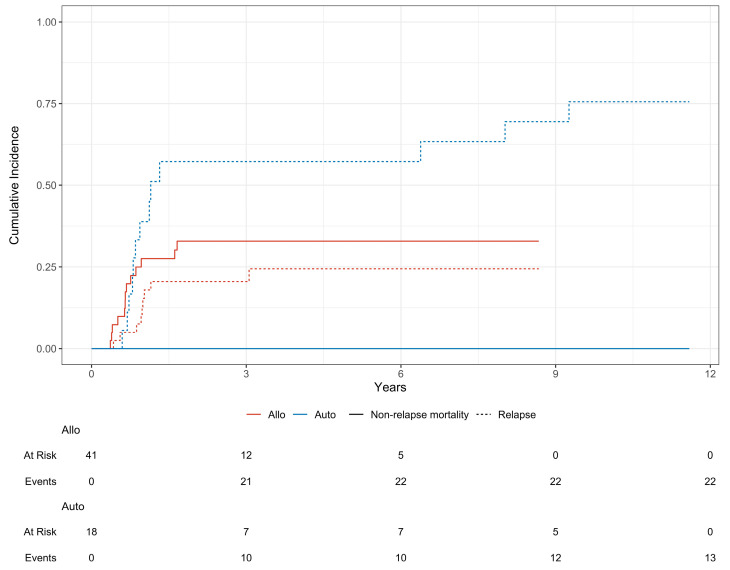
Cumulative incidence of non-relapse mortality (continuous line) and relapse (dotted line) in patients receiving allogeneic transplant (red line) and autologous transplant (blue line) in CR1.

The median EFS was comparable between autologous and allogeneic patients (1.1 vs. 1.7 years—*p* = 0.81). The cumulative incidence of 1-year NRM was 0.00% for autologous and 26% (CI: 13–40) for allogeneic transplantation (*p* = 0.010). The CIR at 1 and 3 years was 39% (CI: 17–61) vs. 16% (CI: 6.2–29) and 57% (CI: 30–77) vs. 21% (CI: 9.7–35), respectively (*p* = 0.005) (Figure 2).

The median survival was not reached in patients treated with TKI in induction, while in the no-TKI group, the median survival was 20 months.

Among the 73 NPM1m patients, the median survival was 11.5 vs. 15.1 months [95% CI: (6.13–20.31); *p* = 0.22], and the median EFS was 11.0 vs. 13.8 months [95% CI: (6.93–16.22); *p* = 0.34] in the no-TKI and TKI groups, respectively. It must be emphasized that the follow-up of no-TKI patients is much longer and dates back from 1999 to 2017, while standard treatment with TKI in induction started in 2018. 

**Figure 3:**
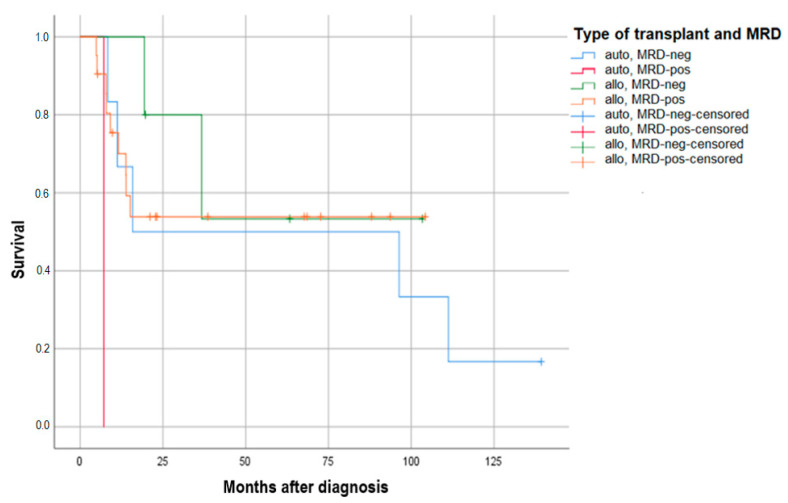
EFS probability at 60 months of the 33 patients FLT3m/NPM1m transplanted in CR1 by type of transplant and MRD status.

Information on pre-transplant MRD status was available for 33 of the 40 patients with NPM1 co-mutation who underwent transplantation in CR1. Of the 33 patients, 11 were MRD-negative by RQ-PCR (6 autologous and 5 allogeneic), while 22 were MRD-positive (1 autologous and 21 allogeneic). The projected 60-month probability of EFS is 54% for patients who received allogeneic hematopoietic stem cell transplantation (HSCT) and were positive for minimal residual disease (MRD) and 50% for patients who received autologous HSCT and were negative for MRD. the comparison between autologous transplantation in MRD-positive and -negative patients is not feasible as only one patient was autografted with positive MRD due to our institutional policy. However, the autologous HSCT survival plots do not demonstrate a plateau in the long term (Figure 3).

**Figure 4:**
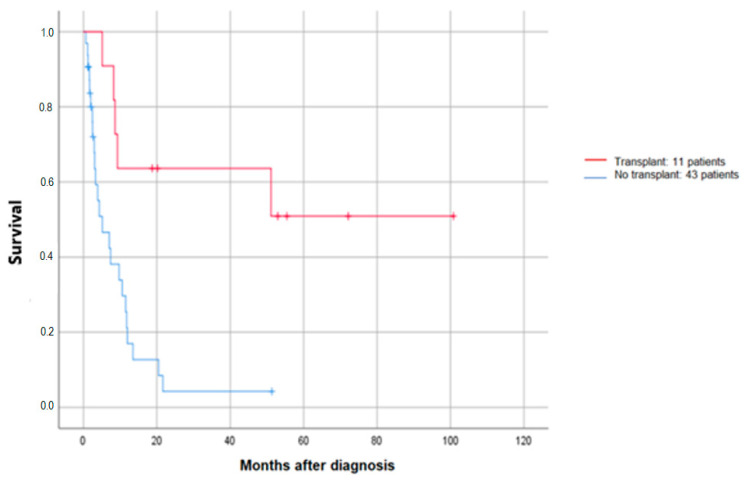
OS probability at 60 months for refractory patients transplanted or not after salvage treatment.

A total of 43 patients (34 who had not received TKI therapy and 9 who had) were found to be refractory to first-line therapy. Twenty-five patients received salvage treatment, with 10 receiving CHT plus TKI, 4 receiving TKI as a single agent, and 11 receiving standard CHT. It is noteworthy that of the 14 patients who received TKI in conjunction with the salvage regimen, 11 underwent allogeneic stem cell transplantation, whereas none of the patients in the standard CHT group underwent transplantation. Figure 4 depicts the OS at 60 months in both transplanted and non-transplanted patients.

Overall, 69 patients received stem cell transplantation, 18 of whom underwent autologous transplantation, and 51 underwent allogeneic transplantation (40 of whom were in CR1, and 11 of whom were refractory).

Preemptive TKI treatment was administered in 8/51 cases with post-transplant FLT3 mutation in hematological CR; all patients are alive in CR at a median of 62 months (range 16–98) post-transplant, and TKI treatment was withheld after 2 years of continuous complete remission.

## 4. Discussion

The introduction of FLT3i has become pivotal in the treatment of FLT3-mutated AML, yet the therapeutic algorithm remains challenging. The current frontline approach includes induction/consolidation chemotherapy followed by oral midostaurin and allogeneic SCT in CR1 in fit patients. Nevertheless, several unanswered questions remain. These include how to optimize the use of different classes and generations of inhibitors to achieve the best response before BMT, the role of MRD in transplant eligibility, and the potential impact of post-transplant maintenance.

In the randomized phase 3 RATIFY trial, CR rates were not significantly different between the midostaurin and placebo arms (58.9 vs. 53.5). However, midostaurin therapy demonstrated a significant prolongation of OS compared to the placebo (74.7 vs. 25.6 months) [4].

In our case series, the presence of a co-mutation of NPM1 and a WBC count higher than 100 × 10^9^/L at diagnosis was found to affect the likelihood of achieving a response. However, in multivariate analysis, only WBCs retained an independent role as a negative prognostic factor. The addition of TKI at the time of induction did not result in a significant difference in the CR rate. However, in univariate analysis, there appeared to be a trend, favoring patients who received TKI. It is likely that the sample size was insufficient to detect differences in this regard, which was also influenced by the presence of the NPM1 mutation. It is noteworthy that an analysis of the reasons why patients who were eligible for transplantation did not undergo this procedure revealed that all early relapses occurred in the group that did not receive TKI. This finding may support the hypothesis that patients treated with TKI have a better quality of response. This finding is consistent with the results of the Quantum-first study, which demonstrated that the proportion of patients achieving undetectable MRD by NGS-PCR assessment was significantly higher in patients treated with the FLT3 inhibitor quizartinib, suggesting the induction of deeper remissions compared to the placebo [21].

The optimal kinase inhibitor for use in frontline treatment is likely to emerge from ongoing randomized phase 3 trials, such as the HOVON and AMLSG cooperative group trial (NCT04027309), which compares gilteritinib with midostaurin following 7+3 backbone chemotherapy.

In our experience, there has been no documented difference in survival between patients who underwent allo-HSCT and those who received high-dose therapy followed by ASCT, regardless of whether or not TKIs were administered in induction. Nevertheless, the CIR is considerably higher in autologous transplant recipients, despite the fact that this group of patients is selected for both the presence of the NPM1 mutation and pre-transplant MRD negativity. Furthermore, our experience indicates that the survival curve of patients who do not receive an allogeneic transplant does not reach a plateau, and late relapses are observed even after six years.

The role of allo-HSCT in FLT3-ITD AML in CR1 has long been controversial [22], further complicated by the 2017 European LeukemiaNet (ELN) risk stratification, which determined the risk of relapse based on the allelic ratio (AR) of FLT3-ITD and the NPM1 co-mutation status. This ambiguity has been resolved by the ELN 2022 guidelines, which state that all patients with FLT3-ITD AML will ultimately fall into the intermediate risk category. This is due to the modifying role of midostaurin-based therapy on FLT3-ITD and the now established prognostic importance of post-chemotherapy MRD. In accordance with the newly established recommendations, all transplant-eligible patients in CR should be considered for allo-HSCT, particularly in the absence of a sensitive marker to monitor MRD [23,24].

To date, the detection of FLT3-ITD MRD in AML has been hindered by its potential instability at relapse, the diversity of patient-specific duplications, and the lack of a standardized method with sufficient sensitivity. For these reasons, current guidelines do not recommend FLT3-ITD MRD monitoring, and it should not be a decision-making factor. Conversely, NPM1 mutations have clear potential for MRD assessment, but only approximately half of patients with FLT3-ITD AML have an NPM1 mutation.

The UK NCRI group conducted a retrospective analysis of 286 patients with FLT3-ITD and NPM1 mutations enrolled in NCRI AML17 and 19 protocols. MRD was available by RQ-PCR for NPM1 in peripheral blood after cycle 2, based on the results of Ivey [25]. Of the 286 patients included in the analysis, 53 were allogeneic in CR1 (32 MRD+ and 21 MRD-). The data suggest that while the benefit of allo-HSCT in CR1 is undeniable in MRD+ patients, it does not add benefit in MRD patients. These results should be interpreted with caution, as they were obtained in patients who had not received first-line TKIs and therefore may be positively selected [26].

Nevertheless, while transplantation remains the standard of care for CR1 patients for the time being, the high relapse rate still observed after allogeneic transplantation raises questions about the efficacy of post-transplant maintenance with TKIs.

The role of post-transplant TKI use has recently been investigated in two prospective randomized trials evaluating the use of gilteritinib and quizartinib. The results of both studies concur that the addition of the inhibitor in the post-transplant setting is efficacious in terms of OS and RFS solely in the cohort of patients who remain MRD-positive pre- or post-transplant. Monitoring for FLT3 is performed using the NGS-PCR method, which is currently considered the most reliable method for determining FLT3 in MRD. However, this method is not yet standardized or widely available [21,27].

In patients with R/R disease, the ADMIRAL trial demonstrated that gilteritinib is an effective bridge to transplantation therapy in terms of tolerability, toxicity, and response rate compared to standard chemotherapy. A two-year follow-up of the same study demonstrated that long-term survival was limited to patients who received an allo-HSCT. However, it is challenging to assess the contribution of transplantation alone to survival, as the majority of transplanted and long-term surviving patients resumed gilteritinib therapy after transplantation [7,28].

Our data appear to be consistent with the findings of Perl et al. [28]. Among the 25 patients undergoing salvage therapy, 14 received TKIs, and 11 of them underwent transplantation. Three of them resumed TKI after transplantation, and the 5-year survival rate was 51%. In contrast, none of the patients who received standard chemotherapy were eligible for transplantation. 

In our experience, we initiated preemptive treatment when we were able to detect the mutation by PCR, with a much higher sensitivity threshold. All eight patients who received this treatment are alive in CR with a median of 62 months after transplantation.

## 5. Conclusions

In conclusion, FLT3-positive acute myeloid leukemia remains an extremely aggressive disease that requires timely treatment and the optimization of the use of new drugs at our disposal to minimize the emergence of resistance. Our findings confirm that FLT3 inhibitors can enhance median survival. Stem cell transplantation remains an important cornerstone in the treatment algorithm for these patients, who seem to miss out on multiple cycles of chemotherapy, during which an increase in the circulating FLT3 ligand has been shown to compete with the action of TKI [29]. However, given the mortality risk of HSCT and the availability of maintenance drugs, it is essential to identify which patients require early transplantation.

The next challenges will be to determine whether there is a category of patients in whom transplantation can be omitted based on MRD and, in parallel, in which patients we should intensify treatment in the post-transplant period. Autologous transplant may be a suitable treatment modality to provide MRD-negative patients with high-dose, short-term treatment, which may overcome the risk of reduced efficacy due to the high levels of FLT3 ligands after multiple cycles of chemotherapy. However, the risk of relapse cannot be underestimated, and only a prospective evaluation on a large sample size of patients treated with TKI in induction may clarify the role of autologous vs. allogeneic transplantation in these patients. 

## Figures and Tables

**Table 1:** Patients’ details.

	All Patients	TKI in Induction		Transplant in CR1		
		No	Yes	Autologous	Allogeneic	*p*
Total patients (M/F)	140 (68/72)	101 (53/48)	39 (15/24)	18 (10/8)	40 (17/23)	
Age (years), median (range)	52 (14–73)	50 (14–73)	54 (27–72)	45 (19–62)	50 (18–65)	0.07
Age > 60 years (%)	38	26 (25%)	12 (30%)	1 (5%)	9 (22%)	0.15
WBC × 10^9^/L at diagnosis, median (range)	50 (0.2–349)	43 (0.22–349)	58 (0.17–260)	24.4 (2.8–95.2)	50.4 (1.6–337)	0.4
Mutation type ITD TKD	120 20	84 17	36 3	12 6	36 4	0.05
NPM1 status Mutated Wild-type	73 67	46 55	27 12	10 8	26 14	0.5
Cytogenetics Favorable Intermediate Unfavorable	4 126 10	2 93 6	2 33 4	2 16 -	2 32 6	-
Midostaurin I line Yes No	39 101	- 101	39 -	2 16	19 21	0.009

**Table 2:** Multivariate analysis.

Characteristic	OR	95% CI	*p*-Value
Sex			
Male	-	-	
Female	1.00	0.45, 2.22	>0.9
Age			
<60	-	-	
≥60	0.45	0.18, 1.06	>0.9
FLT3			
TKD	-	-	
ITD	0.34	0.09, 1.08	0.087
NPM			
Wild-type	-	-	
Mutation	1.96	0.91, 4.30	0.087
GB			
>100,000	-	-	-
≤100,000	3.04	1.23, 7.71	**0.017**

The bold format highlights the statistical significance.

**Table 3:** Reason for not transplanting in CR.

	No TKI	TKI
Non-eligible (age/comorbidity)	9	3
Consolidation CHT + TKI maintenance	4	2
Early relapse	7	0
Refusal	1	1
Toxic death	2	0
Transplant planned	0	3

## Data Availability

The dataset is available on request from the authors.

## References

[1] Gary Gilliland D., Griffin J.D. (2002). The roles of FLT3 in hematopoiesis and leukemia. Blood.

[2] Döhner H., Weber D., Krzykalla J., Fiedler W., Wulf G., Salih H., Lübbert M., Kühn M.W.M., Schroeder T., Salwender H. (2022). Midostaurin plus intensive chemotherapy for younger and older patients with AML and FLT3 internal tandem duplications. Blood Adv..

[3] Kennedy V.E., Smith C.C. (2020). FLT3 Mutations in Acute Myeloid Leukemia: Key Concepts and Emerging Controversies. Front. Oncol..

[4] Stone R.M., Mandrekar S.J., Sanford B.L., Laumann K., Geyer S., Bloomfield C.D., Thiede C., Prior T.W., Döhner K., Marcucci G. (2017). Midostaurin plus Chemotherapy for Acute Myeloid Leukemia with a FLT3 Mutation. N. Engl. J. Med..

[5] Fiedler W., Kayser S., Kebenko M., Janning M., Krauter J., Schittenhelm M., Götze K., Weber D., Göhring G., Teleanu V. (2015). A phase I/II study of sunitinib and intensive chemotherapy in patients over 60 years of age with acute myeloid leukaemia and activating FLT3 mutations. Br. J. Haematol..

[6] Knapper S., Russell N., Gilkes A., Hills R.K., Gale R.E., Cavenagh J.D., Jones G., Kjeldsen L., Grunwald M.R., Thomas I. (2017). A randomized assessment of adding the kinase inhibitor lestaurtinib to first-line chemotherapy for FLT3-mutated AML. Blood.

[7] Röllig C., Serve H., Hüttmann A., Noppeney R., Müller-Tidow C., Krug U., Baldus C.D., Brandts C.H., Kunzmann V., Einsele H. (2015). Addition of sorafenib versus placebo to standard therapy in patients aged 60 years or younger with newly diagnosed acute myeloid leukaemia (SORAML): A multicentre, phase 2, randomised controlled trial. Lancet Oncol..

[8] Perl A.E., Martinelli G., Cortes J.E., Neubauer A., Berman E., Paolini S., Montesinos P., Baer M.R., Larson R.A., Ustun C. (2019). Gilteritinib or Chemotherapy for Relapsed or Refractory FLT3-Mutated AML. N. Engl. J. Med..

[9] Zimmerman E.I., Turner D.C., Buaboonnam J., Hu S., Orwick S., Roberts M.S., Janke L.J., Ramachandran A., Stewart C.F., Inaba H. (2013). Crenolanib is active against models of drug-resistant FLT3-ITD2positive acute myeloid leukemia. Blood.

[10] Altman J.K., Foran J.M., Pratz K.W., Trone D., Cortes J.E., Tallman M.S. (2018). Phase 1 study of quizartinib in combination with induction and consolidation chemotherapy in patients with newly diagnosed acute myeloid leukemia. Am. J. Hematol..

[11] Ferrara F., Barosi G., Venditti A., Angelucci E., Gobbi M., Pane F., Tosi P., Zinzani P., Tura S. (2013). Consensus-based definition of unfitness to intensive and non-intensive chemotherapy in acute myeloid leukemia: A project of SIE, SIES and GITMO group on a new tool for therapy decision making. Leukemia.

[12] Mandelli F., Vignetti M., Suciu S., Stasi R., Petti M.C., Meloni G., Muus P., Marmont F., Marie J.P., Labar B. (2009). Daunorubicin versus mitoxantrone versus idarubicin as induction and consolidation chemotherapy for adults with acute myeloid leukemia: The EORTC and GIMEMA groups study AML-10. J. Clin. Oncol..

[13] Saraceni F., Beohou E., Labopin M., Arcese W., Bonifazi F., Stepensky P., Aljurf M., Bruno B., Pioltelli P., Passweg J. (2018). Thiotepa, busulfan and fludarabine compared to busulfan and cyclophosphamide as conditioning regimen for allogeneic stem cell transplant from matched siblings and unrelated donors for acute myeloid leukemia. Am. J. Hematol..

[14] Luznik L., O’Donnell P.V., Fuchs E.J. (2012). Post-transplantation cyclophosphamide for tolerance induction in HLA-haploidentical bone marrow transplantation. Semin. Oncol..

[15] Meloni G., Proia A., Capria S., Romano A., Trapé G., Trisolini S.M., Vignetti M., Mandelli F. (2001). Obesity and Autologous Stem Cell Transplantation in Acute Myeloid Leukemia. Bone Marrow Transpl..

[16] Noguera N.I., Ammatuna E., Zangrilli D., Lavorgna S., Divona M., Buccisano F., Amadori S., Mecucci C., Falini B., Lo-Coco F. (2005). Simultaneous detection of NPM1 and FLT3-ITD mutations by capillary electrophoresis in acute myeloid leukemia. Leukemia.

[17] Falini B., Mecucci C., Tiacci E., Alcalay M., Rosati R., Pasqualucci L., La Starza R., Diverio D., Colombo E., Santucci A. (2005). Cytoplasmic Nucleophosmin in Acute Myelogenous Leukemia with a Normal Karyotype. N. Engl. J. Med..

[18] Murphy K.M., Levis M., Hafez M.J., Geiger T., Cooper L.C., Smith B.D., Small D., Berg K.D. (2003). Detection of FLT3 Internal Tandem Duplication and D835 Mutations by a Multiplex Polymerase Chain Reaction and Capillary Electrophoresis Assay. J. Mol. Diagn..

[19] Gorello P., Cazzaniga G., Alberti F., Dell’Oro M.G., Gottardi E., Specchia G., Roti G., Rosati R., Martelli M.F., Diverio D. (2006). Quantitative assessment of minimal residual disease in acute myeloid leukemia carrying nucleophosmin (NPM1) gene mutations. Leukemia.

[20] Schuurhuis G.J., Heuser M., Freeman S., Béné M.C., Buccisano F., Cloos J., Grimwade D., Haferlach T., Hills R.K., Hourigan C.S. Minimal/Measurable Residual Disease in AML: A Consensus Document from the European LeukemiaNet MRD Working Party. http://ashpublications.org/blood/article-pdf/131/12/1275/1405674/blood801498.pdf.

[21] Erba H.P., Montesinos P., Kim H.J., Patkowska E., Vrhovac R., Žák P., Wang P.N., Mitov T., Hanyok J., Kamel Y.M. (2023). Quizartinib plus chemotherapy in newly diagnosed patients with FLT3-internal-tandem-duplication-positive acute myeloid leukaemia (QuANTUM-First): A randomised, double-blind, placebo-controlled, phase 3 trial. Lancet.

[22] Bazarbachi A., Bug G., Baron F., Brissot E., Ciceri F., Dalle I.A., Döhner H., Esteve J., Floisand Y., Giebel S. (2020). Clinical practice recommendation on hematopoietic stem cell transplantation for acute myeloid leukemia patients with FLT3internal tandem duplication: A position statement from the Acute Leukemia Working Party of the European Society for Blood and Marrow Transplantation. Haematologica.

[23] Döhner H., Estey E., Grimwade D., Amadori S., Appelbaum F.R., Büchner T., Dombret H., Ebert B.L., Fenaux P., Larson R.A. (2017). Diagnosis and management of AML in adults: 2017 ELN recommendations from an international expert panel. Blood.

[24] Döhner H., Wei A.H., Appelbaum F.R., Craddock C., DiNardo C.D., Dombret H., Ebert B.L., Fenaux P., Godley L.A., Hasserjian R.P. (2022). Diagnosis and Management of AML in Adults: 2022 Recommendations from an International Expert Panel on Behalf of the ELN. Blood.

[25] Ivey A., Hills R.K., Simpson M.A., Jovanovic J.V., Gilkes A., Grech A., Patel Y., Bhudia N., Farah H., Mason J. (2016). Assessment of Minimal Residual Disease in Standard-Risk AML. N. Engl. J. Med..

[26] Thomas I., Dennis M. (2024). Post Induction Molecular MRD Identifies Patients with NPM1 AML Who Benefit from Allogeneic Transplant in First Remission. Blood.

[27] Levis M.J., Hamadani M., Logan B., Jones R.J., Singh A.K., Litzow M., Wingard J.R., Papadopoulos E.B., Perl A.E., Soiffer R.J. (2024). BMT-CTN 1506/MORPHO Study Investigators. Gilteritinib as Post-Transplant Maintenance for AML with Internal Tandem Duplication Mutation of *FLT3*. J. Clin. Oncol..

[28] Perl A.E., Larson R.A., Podoltsev N.A., Strickland S., Wang E.S., Atallah E., Schiller G.J., Martinelli G., Neubauer A., Sierra J. (2023). Outcomes in Patients with FLT3-Mutated Relapsed/ Refractory Acute Myelogenous Leukemia Who Underwent Transplantation in the Phase 3 ADMIRAL Trial of Gilteritinib versus Salvage Chemotherapy. Transplant. Cell Ther..

[29] Levis M. (2011). FLT3/ITD AML and the law of unintended consequences. Blood.

